# Adolescent Anxiety, Depression and Flourishing before and During COVID-19 and the Predictive Role of Baseline Psychological Capital (PsyCap) on Student Mental Health and Subjective Wellbeing During the Pandemic

**DOI:** 10.1007/s10578-023-01568-z

**Published:** 2023-07-07

**Authors:** Jules Finch, Allison M Waters, Lara J Farrell

**Affiliations:** 1 School of Applied Psychology, Griffith University, Gold Coast Campus, Australia; 2 School of Applied Psychology, Griffith University, Mt Gravatt Campus, Australia

**Keywords:** COVID-19, Pandemic, Psychological capital, PsyCap, Anxiety and depression, Flourishing, Subjective wellbeing, Gender, Longitudinal

## Abstract

Studies indicate the COVID-19 pandemic has resulted in rises in adolescent mental health symptoms globally, although the impact of the pandemic on subjective wellbeing is under-researched in this population. Psychological capital (PsyCap), a cluster of four positive psychological constructs comprising hope, efficacy, resilience and optimism (HERO), has demonstrated preventative and promotive qualities on mental health symptoms and subjective wellbeing outcomes with adult populations (employees, university students). However, PsyCap’s influence on these outcomes in young people is unclear. The present exploratory study investigated changes in self-reported anxiety and depressive symptoms (measured via the RCADS-SV) and subjective wellbeing (measured by the Flourishing Scale) from pre-pandemic levels to 3 months into the pandemic and explored gender differences at each time point in a sample of Australian Year 10 students (N *=* 56, *M*age = 14.93 years, *SD* = 0.50, 51.8% male). The longitudinal predictive role of baseline PsyCap on follow-up assessments of anxiety symptoms, depressive symptoms and flourishing were also examined. There were no significant changes in levels of anxiety and depressive symptoms between the timepoints, but flourishing significantly declined from T1 to T2. Baseline PsyCap was not a significant predictor of T2 anxiety and depressive symptoms but was a significant predictor of T2 flourishing. Further, different baseline HERO constructs predicted T2 mental health symptoms and flourishing. Future larger studies building on the current preliminary findings investigating the roles of student PsyCap, mental health and subjective wellbeing are warranted to better understand these constructs in the COVID-19 era and beyond.

## Introduction

COVID-19 was declared a global pandemic on 11th March 2020 [1]. In Australia, as in many other countries, government mandated measures to minimise the migration of COVID-19 meant that frequent hand washing/sanitising, social/physical distancing, mask wearing, lockdowns, travel restrictions and quarantine became commonplace practices and phrases in our everyday lives. Such directives have had significant economic, social and psychological ramifications [2]. For young people, school closures and simultaneous home confinement was a unique containment method that disrupted students’ learning environments, access to educational resources, peer interactions, physical activities, and general daily routine. A 2021 report by the Organisation for Economic Co-operation and Development (OECD) comprising data from its member countries, found that in young people (aged 15 to 24) mental health issues had at least doubled in the period 2020 to 2021, and that school closures resulted in the deterioration of many protective factors (i.e., daily routines, social contact, social and emotional support, sense of belonging and connectedness to a community). The report concluded that “the COVID-19 crisis has turned into a mental health crisis for young people” [3].

### The Impact of COVID-19 on Youth Mental Health Outcomes

Early substantive reviews of the global empirical literature seeking to understand the impact of the COVID-19 pandemic on mental health outcomes in youth indicated anxiety and depression appeared to be key areas of compelling concern [4, 5]. A meta-analysis of the global prevalence of depressive and anxiety symptoms in children and adolescents during COVID-19 found across 29 samples (N *=* 80,879), 1 in 5 (20.5%) youths reported experiencing clinically elevated anxiety, and 1 in 4 (25.2%) youths reported experiencing clinically elevated depression; rates that are estimated to be double pre-pandemic levels [6]. Indeed COVID-19 has been found to be associated with a host of other mental health difficulties, including obsessive-compulsive symptoms [7, 8], social anxiety [9] and suicidality [10]. Pandemic-driven mental health concerns in youth have persisted beyond vaccination programs and recovery from the pandemic [11]. Identified risk factors for poorer mental health outcomes in young people during the pandemic included low family income [e.g., 12], family stress/conflict [e.g., 12, 13], pre-existing mental health concerns [e.g., 4, 6, 8], adolescence [e.g., 14, 15, 4] and female gender [e.g., 14, 4]. While it is necessary to understand which youths experience distress during the pandemic, it is equally important to understand potential factors that promote flourishing during these challenging times. A key area advocated for further investigation in context to COVID-19 and development, is the evaluation of young people’s wellbeing [9].

### Youth Subjective Wellbeing

Subjective wellbeing (SWB) is a multidimensional construct comprising hedonic (e.g., happiness, satisfaction with life) and eudaimonic (e.g., purpose/meaning in life) constituents [16]. The concept of SWB has been paramount in the assessment of factors that enable individuals, organisations and nations to flourish [17–19]; whereby flourishing describes high levels of subjective wellbeing [20]. SWB during COVID-19, and in particular, the ability to thrive despite the challenges and threats of an ongoing global pandemic may be attributable to post-traumatic growth. That is, the ability of an individual to experience positive psychological changes after significant adversity [21]. Indeed, a recent (2022) study on post-traumatic stress and post-traumatic growth in the aftermath of COVID-19 (N *=* 412, aged 9 to 20 years, 54.8% male, *M*age 15.12, *SD* = 2.15), revealed the majority (68.9%) of children and adolescents reported a high level of post-traumatic stress symptoms as a result of the pandemic, however, 39.8% of this majority also experienced post-traumatic growth [22]. This study highlights the significance of assessing psychological change during the pandemic from both negative and positive perspectives. Considering the social, emotional and learning outcomes for youths during the COVID-19 pandemic [e.g., 23, 4, 24, 12], understanding the impact of the pandemic on mental health symptoms *and* SWB in adolescent students is an important avenue of inquiry. More so given mental health, SWB and the school context are inextricably interwoven [25].

To date, only one Australian study conducted by Magson and colleagues (2020) has examined the longitudinal impact of the COVID-19 pandemic on adolescents’ mental health and SWB outcomes, including potential risk and protective factors. Magson et al.’s study conducted in New South Wales (N *=* 248, 51% girls, *M*age 14.4, *SD* = 0.5) measured anxiety, depression, and life satisfaction in the year before COVID-19 (2019) and again 2 months into the pandemic during lockdown, when students had moved to online learning (May 2020). The authors’ findings indicated adolescents experienced significant increases in anxiety and depression from pre-pandemic (Time 1) to during the pandemic (Time 2) and these increments were higher in girls than boys. Findings also illustrated significant decline in life satisfaction over the same period, again the effect was more pronounced in girls. Further, COVID-19 related stress, difficulties with online learning, and increased conflict with parents predicted increases in anxiety and depression from Time 1 to Time 2. Compliance to stay-at-home mandates and feeling socially connected during lockdown were found to be protective factors against poorer mental health [26].

Given the empirical support for the significant negative impact of COVID-19 on the mental health and SWB of young people, the importance of ongoing evaluation of these outcomes and the quest to identify risk and protective factors that might help reduce mental health symptoms and promote SWB in youth is much needed during the pandemic and beyond [6, 26, 27]. One worthy avenue of research which has gained some empirical attention during the COVID-19 pandemic is the construct of psychological capital [28–30].

### Psychological Capital

Psychological capital (PsyCap) comprises four positive psychological resources: hope, (self-) efficacy, resilience and optimism (aka ‘HERO’) and has been studied in adult samples within the field of positive organisational behaviour for the past two decades [31, 32]. The PsyCap model is based on theoretical frameworks from positive psychology and stress and adaptation research. Specifically, PsyCap subscribes to Fredrickson’s ‘broaden-and-build’ theory positing that experience and awareness of positive emotions broadens an individual’s thought-action repository, from which psychological resources are built [33, 34]. Also foundational to the PsyCap model is Hobfall’s (2002) ‘conservation of resources’ theory which advocates that developmental processes tend to generate “resource caravans” [35]. These caravans travel together and work in a synergistic manner generating “differentiated manifestations over time and across contexts” [32].

Conceptualisation of hope [36], self-efficacy [37], resilience [38], and optimism [39] as generalised constructs has prompted some support for PsyCap as a universal/non-domain specific construct [40]. Nonetheless, and perhaps due to its positive organisational behaviour origins, PsyCap in the main, is a contextualised construct. That is, most empirical studies have been conducted in the workplace with employees and PsyCap has been studied in numerous occupational sectors focusing on outcomes of workforce productivity, attitudes, behaviours, health, relationships and wellbeing [32]. Additionally, intervention studies have demonstrated that PsyCap can be taught and developed in adults [41, 42] and that its development and impacts are generalisable to Western and non-Western populations [43]. Similarities in contextual features of workplaces and educational institutions (e.g., governance, infrastructure, citizenship, relationships, performance, and appraisal) has inspired researchers to examine PsyCap in students using similar outcome measures to employees. For example, research in university samples has investigated PsyCap and outcomes of academic engagement [e.g., 44], academic performance [e.g., 45], mental health symptoms [46] and flourishing [29], providing support for positive outcomes associated with PsyCap in young adult learners.

#### PsyCap and the School Context

More recently PsyCap research has been extended to youth within a school context via cross-sectional studies, with initial findings indicating negative relationships between PsyCap and mental health symptoms [i.e., anxiety and depression; 47, 48], and positive relationships between PsyCap and student wellbeing [47–51]. In applying PsyCap research to youth, it is recognised that adjustments should be made for the developmental nuances of young people compared to adults. Accordingly, a recent cross-sectional study examining PsyCap in students (N *=* 456, *M*age 11.54, *SD* = 1.20, 47% female) integrated a developmentally tailored social-ecological framework of resilience to the PsyCap model [47]. The study, the first to evaluate PsyCap in Australian children and adolescents, found that PsyCap was a significantly stronger predictor of flourishing, anxiety symptoms and depressive symptoms, than any of the individual HERO constructs; suggesting that the *combined* positive psychological resources of PsyCap might be a buffer to mental health issues and promote SWB in youth [47].

Given the profoundly negative impact of COVID-19 on the mental health of youths, research examining positive psychological resources as potential predictors of common student mental health concerns and SWB during the pandemic era is of high significance. Whilst studies of youth PsyCap remain relatively scarce, there has been several recent studies investigating PsyCap during COVID-19 among young adults. For example, a recent US longitudinal study of university students (N *=* 609, *M*age = 27.36, *SD* = 9.91) found there was a shift in the predictive roles of HERO constructs from before COVID-19 to after the onset of the pandemic on wellbeing; with the strongest predictors before the pandemic being self-efficacy and resilience, changing to hope and optimism during COVID-19 [29]. These findings reflected a key theoretical principle of the PsyCap model, in regard to the synergistic nature of positive psychological resources, creating “differentiated manifestations over time and across contexts” [32]. Further, another study conducted in China following the onset of COVID-19 demonstrated significant relationships between PsyCap and social anxiety (*r* = − .42, *p* < .05) among older youth and young adults [N = 600, aged 18–22; 28]. Moreover, an initial evaluation of a brief, school-based intervention program designed to develop HERO capabilities in adolescents during COVID-19 found longitudinal support for the development of HERO constructs in young people during their final year of high school, which was significantly disrupted by COVID-19 [52]. Further empirical investigations of PsyCap in youth may be useful for policymakers, schools, researchers, and clinicians seeking to understand protective factors and develop interventions that might mitigate risk of mental health problems and promote wellbeing during this time of significant risk and burden for young people.

### The Current Study

The current study is an exploratory evaluation of mental health symptoms (anxiety and depressive symptoms) and SWB (flourishing) at a pre-pandemic juncture and 3-months later, following the onset of the pandemic in Australian adolescent students aged 14 to 17 years, including examining gender differences. Building on previous findings for the cross-sectional associations and predictive role of PsyCap on adolescent students’ mental health [53, 54] and SWB [47, 49, 50], the longitudinal predictive role of PsyCap on variance in mental health and SWB is tested by examining baseline PsyCap (before the pandemic) on follow-up assessments of anxiety and depressive symptoms and flourishing (during the pandemic). Specifically, the following hypotheses are evaluated:

H1: Anxiety and depressive symptoms will significantly increase from pre-pandemic (Time 1) to during the pandemic 3-months later (Time 2) in adolescent students.

H1a: Further, there will be a significant main effect of gender on mental health (significantly higher anxiety and depressive symptoms in girls than boys at both time points), and

H1b: There will be a significant time X gender interaction effect (greater increase in symptoms in girls compared to boys).

H2: Levels of flourishing will significantly decrease from Time 1 to Time 2, and

H2a: Further, there will be a significant main effect of gender on subjective wellbeing (significantly lower flourishing for girls than boys across time), and

H2b: There will be a significant time X gender interaction effect (greater decrease in levels of flourishing in girls compared to boys)..

The present study also seeks to answer the following novel research question:

Will pre-pandemic baseline PsyCap predict anxiety and depressive symptoms and flourishing 3 months later, during the COVID-19 pandemic in adolescent students?

## Method

### Participants

Participants in the current study were Year 10 students from a Christian co-education independent (private) school in South-East Queensland, aged 14 to 17 years who were part of a larger PsyCap research project examining student PsyCap, mental health symptoms, subjective wellbeing and learning outcomes. Of 105 students in the grade, 100 obtained parental consent to participate. Overall, a total of 95 students participated in the study (54.7% male, *M*age = 14.93, *SD* = 0.44). After data screening (see details further below), the final sample of participants who had completed both time points was 56 (53% of the entire grade, 51.8% self-identified as male, 48.2% as female, 0% as other, *M*age = 14.93 years, *SD* = 0.50). The majority of participants were born in Australia (69.6%) with the remaining born in the United Kingdom (8.9%), South Africa (5.4%), New Zealand (5.4%), China (5.4%), Vietnam (3.6%) and Korea (1.8%). Most (78.6%) lived with both parents, 7.1% shared between mother and father, 3.6% with mother only, 3.6% with father only, 3.6% with their aunt and uncle and 3.6% with a homestay family. The majority spoke English as their primary language at home (92.9%). Other primary languages were Chinese (3.6%), German (1.8%) and Korean (1.8%).

### Procedure

#### Ethical Approval

was granted from the affiliated university’s Human Research Ethical Committee (ref: GU2019/801). Data were collected at Time 1 (T1) on 27th February 2020 as part of the wider PsyCap research study. Following the announcement of COVID-19 as a global pandemic on 11th March 2020, most schools in Australia were closed from late March until May 2020, and the high school in this study closed for 6 weeks during that time. Within Queensland, the Department of Education placed an embargo on all state (public) school research severely impacting data collection for approved studies. The participating independent (i.e., non-public) school agreed to extend their engagement in the research to examine the impact of COVID-19 on their Year 10 students’ mental health and subjective wellbeing, relative to pre-pandemic levels. A variation to ethics was submitted for this purpose and approved. A second set of data were collected at Time 2 (T2) on 9th June 2020, two weeks after schools re-opened following lockdown. Consent was obtained via the school emailing a letter to parents detailing the study. One hundred parents consented to their child’s participation. Parental consent for the second round of data collection was obtained via the same method as T1 data collection. As part of the survey instructions at both timepoints, students were informed that participation is voluntary, and they can either not start the survey or withdraw their participation at any time without question or penalty. At both timepoints, the online survey was accessed and completed on individual student laptops at school during a single period of class time (50 min). Data were collected on Limesurvey software and transferred to IBM SPSS Version 28 for analysis.

### Measures

#### Demographic Background

Demographic information including age (at last birthday), gender (male, female, other), country of birth, primary language spoken at home, and living arrangements, were gathered.

#### Psychological Capital

PsyCap was evaluated via the Measure of Youth-Hope, Efficacy, Resilience and Optimism (MY-HERO), an instrument developed for measurement of PsyCap within a school context for students from Grades 4 to 12. Scale development protocols [55] were followed including pilot work (tool conceptualisation, scale construction and try-out with a focus group of children from public and private schools). A scale development study (N *= >* 600) paper is currently in preparation for publication to ensure this tool is accessible for future research and community use. The MY-HERO scale is a 63-item measurement tool assessing self-reported levels of hope (10 items), self-efficacy (27 items), resilience (15 items) and optimism (11 items) in school-aged students. Items for each subscale of MY-HERO include adapted items from psychometrically robust children and youth measures of hope [Children’s Hope Scale; 56], self-efficacy [Self-Efficacy Scale for Children; 57], social – ecological resilience [Children and Youth Resilience Measure; 58] and optimism [optimism subscale of the Youth Life Orientation Test; 59]; revised for the contextual requirements of an educational setting. Items were added, and unsuitable items (e.g., items with low face validity) were removed to ensure a final measure that sufficiently represented PsyCap in a school environment. For example, for hope, each of the six Children’s Hope Scale items was re-written for the school context (i.e., “I am doing just as well as other kids my age” was changed to “In school I am doing just as well as other kids my age”). Four additional items were created to capture goal-oriented agency (e.g., “If I set a goal at school, I believe I will find the best way to achieve it”) and goal-oriented pathways (e.g., “At school, there is more than one way to achieve my goals”). For items on the hope, resilience, and optimism scales, participants are asked to read each statement and rate their level of agreement. Responses were rated on a six-point scale ranging from 1 (strongly disagree) to 6 (strongly agree). For self-efficacy, participants are presented with statements that reflect competencies at school (e.g., “I can be honest with my teachers”, “I can concentrate on school subjects during class”) and asked to rate how well they can do each thing. Response items were rated on a six-point scale where 1 = “I can’t do this”, 2 = “I probably can’t do this”, 3 = “I possibly can do this”, 4 = “I probably can do this”, 5 = “I most likely can do this” and 6 = “I definitely can do this”. Scores are summed to give scale totals for hope from 10 (lowest possible score) to 60 (highest possible score), for efficacy (from 27 to 162), for resilience (from 15 to 90), for optimism (from 11 to 66), and an overall PsyCap total (from 63 to 378). Studies utilising the MY-HERO measure [e.g., 52] have reported good to excellent internal reliability subscale coefficients (i.e., *α* = 0.84 to.93). Cronbach’s alphas for the current study were *α* = 0.90 (hope), *α* = 0.94 (efficacy), *α* = 0.87 (resilience) and *α* = 0.92 (optimism).

### Mental Health Symptoms

Anxiety and depression symptoms were assessed via the Revised Children’s Anxiety and Depression Scale–Short Version [RCADS-SV; 60]. The RCADS-SV is a 25-item self-report measure comprising 10 items capturing depression (e.g., “I feel sad or empty”) and 15 items which tap a ‘broad anxiety’ factor [60]. Included in the anxiety factor are items representing five subtypes of anxiety: social phobia (e.g., “I worry what other people think of me”), separation anxiety (e.g., “I am afraid of being in crowded places”), generalised anxiety (e.g., “I worry that something bad will happen to me”), obsessive compulsive tendencies (e.g., “I have to do some things over and over again”), and panic (e.g., “I suddenly start to tremble or shake when there is no reason for this”). Participants are asked to indicate how often these things happen to them. Responses are given on a 4-point scale: 0 = “never”, 1 = “sometimes”, 2 = “often” and 3 = “always”. Total scale scores range between 0 (lowest possible score) to 75 (highest possible score). Normative data for a school-based sample (N *=* 1060) organised by gender and grade level indicated *M*total = 17.20 (*SD* = 8.53) for boys (N *=* 102); and *M*total = 19.45 (*SD* = 8.63) for girls (N *=* 170) across 9th and 10th grade cohorts [60]. The anxiety and depression subscales of the RCADS-SV has demonstrated good internal consistency in previous school samples with *α* = 0.83 and *α* = 0.86 for the anxiety subscale and α = 0.80 for the depression subscale [47, 60]. In the current study the total scale reliability coefficient alphas at T1 and T2 respectively were 0.96 and 0.95.

### Subjective Wellbeing

Student SWB was evaluated using the 8-item Flourishing Scale [FS; 61]. Items include, “I lead a purposeful and meaningful life” and “I am engaged and interested in my daily activities”. Responses are rated on a seven-point scale from 1 (strongly disagree) to 7 (strongly agree). Scores are summed to give an overall total between 8 (lowest possible score) to 56 (highest possible score). In previous school-based PsyCap studies, the FS has demonstrated good internal consistency of 0.84 and 0.88 [47, 50]. In the current study, the Cronbach’s alphas were *α* = 0.91 (T1) and *α* = 0.94 (T2).

### Analytic Plan

A priori power analysis was performed prior to T2 data collection using G*Power 3.1 [62] to determine sample size required for the main analysis. With effect size set to 0.3, error rate of 0.05 and power set to 0.80, a sample size of 24 was indicated for the analysis of variance ANOVA and 49 for regression analysis (with four predictors). All significance testing were performed with *α* set at 0.05 (two tailed). The ANOVA determined the effects of gender and time on anxiety, depression and flourishing; differences between genders; and over time. Correlational analysis was conducted to ascertain strength and direction of relationships between the variables of interest. Hierarchical regression analyses were performed to test the predictive role of MY-HERO constructs on variance in outcome measures of mental health symptoms and SWB during the pandemic (T2) after controlling for T1 outcomes. Descriptive statistics were calculated. Reliability analyses were conducted for all measures to establish internal consistency (Cronbach’s alpha) and only scales or subscales with acceptable reliability statistics (*α* > 0.70) were included in analyses.

## Results

### Data Screening

At T1, 85 online surveys were commenced including three pen and paper surveys (due to technical difficulties with logging onto the online version). Paper survey responses were inputted into the dataset by the first author. Within the T1 dataset, there were seven duplicate cases (participants starting another survey after completion of first); one case with demographic information and no scale responses, and one case with no information at all; these nine cases were deleted. A missing values analysis indicated that of the remaining 76 cases; six were missing > 10% of responses; including five participants who did not provide responses to mental health symptoms and wellbeing, thus these were also deleted. Little’s MCAR test determined that the remaining 0.29% data points missing were missing completely at random [Little’s MCAR test: *X*^*2*^ (364) = 160.04, *p* = 1.00]. Expectation maximisation (EM) was used to impute values for the missing data, resulting in a final T1 sample N *=* 70. At T2, 89 online surveys were recorded including 10 cases where no responses were provided for symptoms and wellbeing; derived from six participants. These cases were deleted leaving a T2 final sample of N *=* 79 with no missing data. The datasets from T1 and T2 were merged, and a final sample of paired responses (N *=* 56) were used for analysis.

Inspection of z-scores revealed two extreme data points > ± 3.29 in one case: one on the MY-HERO Hope scale (-3.42) and the other on T1 Flourishing Scale (-3.48). Visual inspection of the individual participant responses indicated these represented genuinely unusual data points and were retained for analysis. Sensitivity analysis run with and without this case demonstrated no substantial changes to outcomes and thus the case was kept in the final dataset for completeness. Assumptions of normality required for ANOVA were tested and met. Specifically, anxiety, depression and flourishing at both timepoints were normally distributed, as assessed by Normal Q-Q plots; assumption of homogeneity of variances as determined by Levene’s test was met (*p*s > 0.05) for all dependent variables (DVs) across all levels of independent variables (IVs); and Box’s test of equality of covariances matrices confirmed there was homogeneity of covariances (*p*s > 0.05). Visual inspection of histograms, P-P plots and scatterplots confirmed assumptions of distribution, linearity, homogeneity and homoscedasticity were met for regression analyses.

### Correlations, Descriptive Statistics and Internal Consistency

To examine the strength and direction of relationships among scores of hope, efficacy, resilience, optimism, anxiety and depression symptoms, and flourishing, Pearson’s correlation coefficients were produced via bivariate correlation analyses. Due to the sample size, bootstrapping (1000 samples) was performed to ensure robust estimates of means, standard errors and confidence intervals for correlation coefficients. Means, standard deviations, correlations and internal consistency for scales are presented in Table 1.

**Table 1:** Means, Standard Deviations, Internal Reliabilities and Correlation Coefficients for all Variables (N = 56)

Variable	1	2	3	4	5	6	7	8	9
1. T1 Hope	(0.90)	0.71^**^	0.77^**^	0.61^**^	0.84^**^	− 0.52^**^	− 0.48^**^	0.68^**^	0.45^**^
2. T1 Efficacy	-	(0.94)	0.64^**^	0.68^**^	0.93^**^	− 0.62^**^	− 0.65^**^	0.59^**^	0.37^**^
3. T1 Resilience	-	-	(0.87)	0.75^**^	0.86^**^	− 0.49^**^	− 0.49^**^	0.60^**^	0.26
4. T1 Optimism	-	-	-	(0.92)	0.84^**^	− 0.61^**^	− 0.52^**^	0.56^**^	0.25
5. T1 PsyCap	-	-	-	-	(0.97)	− 0.66^**^	− 0.64^**^	0.68^**^	0.38^**^
6. T1 Anx/Dep	-	-	-	-	-	(0.96)	0.84^**^	− 0.53^**^	− 0.29^*^
7. T2 Anx/Dep	-	-	-	-	-	-	(0.95)	− 0.49^**^	− 0.33^*^
8. T1 Flourishing	-	-	-	-	-	-	-	(0.91)	0.24
9. T2 Flourishing	-	-	-	-	-	-	-	-	(0.94)
Mean	45.88	127.05	69.47	46.20	288.59	20.70	21.89	45.52	42.00
SD	6.97	20.50	9.79	9.07	40.89	15.32	15.49	7.60	10.92

Note.^**^p < .01, ^*^p < .05. Reliability coefficients on the diagonal. Anx/Dep = anxiety and depression symptoms

### Anxiety and Depression Symptoms Across Time and Gender

**Fig. 1:**
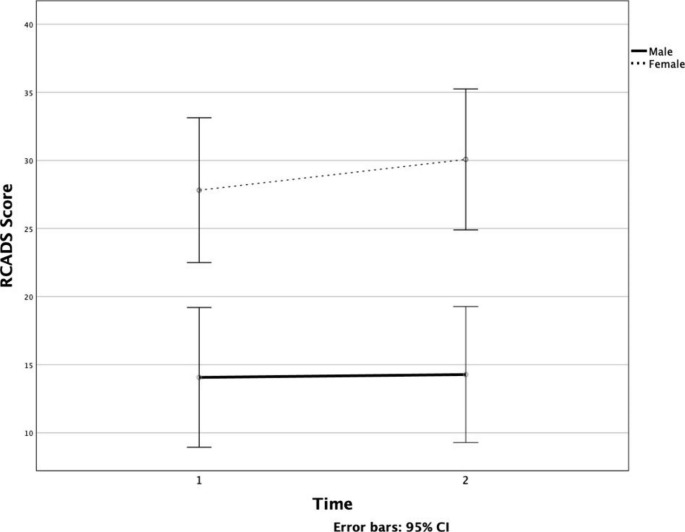
Line Graph with Error Bars Depicting the Mean Change in Anxiety and Depression Symptoms at T1 and T2 by Gender

A 2 (time) x 2 (gender) mixed factorial ANOVA was conducted to analyse main and interaction effects of time and gender on anxiety and depression symptoms. There was no significant effect of time on overall anxiety and depression symptoms, *F*(1,54) = 1.11, *p* = .30, partial $\eta$^2^ = 0.02 (Fig. 1). There was a significant main effect for gender in levels of anxiety and depression symptoms, *F*(1, 54) = 18.42, *p* = < 0.001, partial $\eta$^2^ = 0.25, with girls overall reporting higher levels of anxiety and depression symptoms than boys at both time points (see Table 2). There were no significant time X gender interactions on outcomes of anxiety and depression symptoms, *F*(1, 54) = 0.77, *p* = .39, partial $\eta$^2^ = 0.01.

**Table 2:** Mean Scores and Standard Deviations for Anxiety and Depression Symptoms, and Flourishing by Gender at Both Timepoints (N = 56)

		Males (*n* = 29)					Females (*n* = 27)	
		*M*	*SD*			*M*		*SD*
Anx/Dep	T1	14.07	11.71			27.81		15.70
	T2	14.28	12.04			30.07		14.74
Flourishing	T1	46.83	7.04			44.11		8.05
	T2	42.69	11.50			41.26		10.42

*Note.* Anx/Dep = anxiety and depression symptoms

### Flourishing Across Time and Gender

**Fig. 2:**
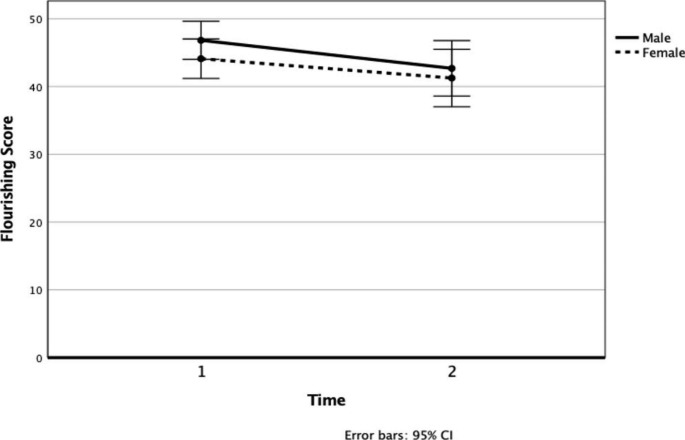
Line Graph with Error Bars Depicting the Mean Change in Levels of Flourishing at T1 and T2 by Gender

A 2 (time) x 2 (gender) mixed factorial ANOVA was conducted to analyse main effects and interaction effects of time and gender on flourishing. There was a significant main effect of time on flourishing, *F*(1,54) = 4.91, *p* = .03, partial $\eta$^2^ = 0.08 with flourishing significantly decreasing from T1 to T2 (Fig. 2). There was no significant main effects for gender in flourishing, *F*(1, 54) = 1.11, *p* = .30, partial $\eta$^2^ = 0.02. There was no significant time X gender interaction on flourishing, *F*(1, 54) = 0.17, *p* = .69, partial $\eta$^2^ = < 0.01.

### Baseline PsyCap Predictor of Time 2 Mental Health Symptoms and SWB

Hierarchical regression analyses were performed to investigate whether mental health symptoms and SWB at T2 could be predicted by baseline PsyCap predictors. In the first step, T1 mental health (anxiety and depression symptoms) or SWB (flourishing) were entered. In the second step, HERO predictors (hope, efficacy, resilience and optimism) were entered. Results of the regression models are presented in Table 3.

**Table 3:** Hierarchical Regression Predicting T2 Anxiety and Depression Symptoms and Flourishing From Baseline HERO (N = 56)

Predictor	T2 Anx/Dep		T2 Flourishing	
	𝛽	*R* ^*2*^ *∆*	𝛽	*R* ^*2*^ *∆*
Step 1		0.70^***^		0.06
Time 1 Dependent Variable	0.84^***^		0.24	
Step 2		0.04		0.18^*^
Hope	0.14		0.58^*^	
Efficacy	− 0.28^*^		0.16	
Resilience	− 0.17		− 0.24	
Optimism	0.17		0.03	

Note.^***^p < .001, ^**^p < .01, ^*^p < .05. Anx/Dep = anxiety and depression symptoms

#### Anxiety and Depression Symptoms

The first hierarchical regression analysis predicted T2 anxiety and depression symptoms. The first step included T1 anxiety and depression symptoms which was a significant predictor of T2 anxiety and depression symptoms, accounting for 70% of the variance in T2 overall anxiety and depression symptoms, *F*(1, 54) = 127.69, *p* < .001. The second step accounted for a further 4% of variance in T2 anxiety and depression symptoms, though this was not statistically significant, ∆*F*(4, 50) = 2.00, *p* = .11. Of the HERO variables, baseline efficacy was a significant predictor of T2 anxiety and depression symptoms, *t*(50) = -2.41, *p* = .02, *sr*^*2*^ = 0.03; demonstrating those higher in efficacy at baseline were lower in anxiety at T2, whereby baseline efficacy accounted for 3% of their T2 anxiety and depression symptom after controlling for their T1 anxiety and depression symptoms.

#### Flourishing

The second hierarchical regression analysis predicted T2 flourishing. The first step consisted of T1 flourishing, which was not a significant predictor of T2 flourishing and accounted for only 6% of variance in T2 flourishing, *F*(1,54) = 3.29, *p* = .08. Overall, the second step accounted for a further 18% of the variance in T2 flourishing and this was significant. ∆*F*(4, 50) = 2.94, *p* = .03. Of the baseline HERO variables, hope was a significant predictor of T2 flourishing, *t*(50) = 2.56, *p* = .01, *sr*^*2*^ = 0.10; demonstrating that those students who were higher in hope at baseline were higher in flourishing at T2, with baseline hope accounting for 10% of T2 flourishing.

## Discussion

The aim of this study was to investigate levels of mental health symptoms and subjective wellbeing (SWB) in adolescent students 2-weeks before COVID-19 was declared a global pandemic and again 3-months later during the pandemic after a 6-week period of school closures and home confinement. The impact of gender and time on changes between baseline and follow-up levels were also explored. Additionally, predictive relationships between pre-pandemic baseline positive psychological resources of hope, efficacy, resilience, and optimism on 3-month follow-up outcomes of mental health symptoms and SWB were examined.

### Student Mental Health Symptoms

Results indicated there were no significant changes in overall anxiety and depression symptoms from baseline (pre-pandemic) to follow-up (during the pandemic) for the overall cohort and within gender groups, and therefore the hypotheses H1 and H1a were not supported. These results contrast with Magson et al.’s (2020) findings of significant increases in levels of anxiety and depression in Australian adolescent students from pre-pandemic to during the pandemic. The comparatively smaller sample of the current study, impacting power to detect significant effects (see limitations below) may be a key factor for this distinction. However, given the convergence for findings of SWB in both studies, other explanations warrant exploration. For example, differences in findings may also be due to students’ levels of mental health symptoms being elevated when measured during a period of school closures and learning from home [26], compared to when the students had returned to school, as was the case in the current study. This may indicate that online learning at home due to COVID-19 school closures may have more negatively impacted students’ mental health compared to when they were back at school. Alternatively, these differences in findings might suggest that mental health symptoms were relatively stable across time in the current sample of adolescents compared to the sample from the previous study. The findings of significant differences in levels of anxiety and depression symptoms between gender groups at both timepoints (higher for girls), aligns with the pre-COVID-19 and COVID-19 literature that female gender is a risk factor for increased symptoms of anxiety and depression [6, 25]. Additionally when compared to the normative data for anxiety and depression symptoms (i.e., total RCADS-SV score) by gender and year group [60], female students in the current sample reported higher levels of overall anxiety and depression symptoms than the normed sample at T2 (i.e., greater than 1 SD from the normative mean). This finding warrants further investigation to establish whether there may be a more universal change in the general population of female adolescents or if change was specific to this cohort. This is particularly pertinent in context to the timing of this study being in the relatively early stages of the pandemic, and that meta-analytic research has indicated that the prevalence of clinically elevated anxiety and depressive symptoms were higher in studies conducted later into the pandemic, particularly in girls [6]. Further longitudinal evaluation of anxiety and depression symptoms may provide clearer indications of the longer-term impact of COVID-19 on mental health symptomology in young people.

### Student Subjective Wellbeing

There was a significant decrease in levels of flourishing from baseline to follow-up for the overall cohort, supporting hypothesis H2. This supports the findings of Magson et al’s (2020) study that measured SWB (indexed by life satisfaction) before and during COVID-19 and their findings of a significant decline in the outcome between timepoints [26]. However, unlike Magson et al.’s study, there were no significant gender differences on the outcome of flourishing and therefore hypothesis H2a is not upheld. The current finding might suggest that flourishing may develop and be affected in similar way for males and females, or that the unique factors of the pandemic impacted boys’ and girls’ flourishing in the same way in the current sample, particularly as they are from the same school and grade cohort. Surprisingly, T1 flourishing did not significantly predict T2 flourishing, implying that SWB might be much less stable than mental health outcomes in young people. Given the study design (i.e., the absence of a control group who did not experience the same pandemic conditions), it is unknown whether the drop in levels of flourishing was directly related to COVID-19. Certainly, it is conceivable that all students’ sense of SWB substantially declined in context to the challenges they faced due to COVID-19 (e.g., unprecedent government directives, learning from home, isolation from friends and teachers) and the uncertainty they continued to navigate (even after some restrictions lifted) given the ever-evolving nature of the global pandemic. The current findings suggest that flourishing may be an important aspect of youth development that requires evaluation during COVID-19 and beyond. Given the relatively brief follow-up period of this study, in context to the ongoing and escalating impacts of COVID-19, it may be that decreases in SWB are an early marker of risk for longer-term symptoms. Longer term follow-up of student’s mental health symptoms would elucidate whether reduced flourishing is indeed a predictor of poorer long-term mental health among adolescents.

### Predictive Role of PsyCap

PsyCap (combined HERO constructs) did not significantly predict T2 anxiety and depression symptoms after controlling for T1 symptoms. However, baseline student (self-) efficacy was a significant predictor of T2 anxiety and depression symptoms demonstrating that those students with stronger beliefs in their capabilities to perform school-related (i.e., social, emotional, and learning) activities before the pandemic experienced lower levels of mental health symptoms during the pandemic, after controlling for baseline symptoms.

PsyCap was a significant predictor of T2 flourishing after controlling for T1 flourishing; indicating that higher levels of PsyCap pre-pandemic significantly and positively impacted adolescent students’ SWB during the pandemic. Additionally, of the HERO constructs, baseline hope was a statistically significant predictor of follow-up flourishing; illustrating that students’ pre-pandemic capacity to set school-based goals, create pathways and generate the motivational momentum to achieve those goals was a vital resource to their sense of subjective wellbeing during the pandemic. Overall, the significant findings that students higher on indices of HERO at baseline prior to the pandemic fared better on measures of mental health symptoms and SWB 3-months later (during the pandemic), after controlling for T1 symptoms, provide a promising empirical rationale to develop interventions seeking to cultivate HERO resources as a mechanism by which to reduce mental health symptoms and improve SWB.

### Limitations and Future Research Directions

Two key limitations to the present study are convenience sampling and sample size; factors influenced by the declaration of a global pandemic and the suspension of state school research in Queensland, Australia during 2020. This rendered the study with relatively small samples at both time points from a single site. Although bootstrapping was performed, the limited sample from one metropolitan private Christian school cohort could be problematic in regard to generalisation of findings to youth more broadly. Missing data at both timepoints further reduced the overall sample size for longitudinal analysis, impacting statistical power, which may explain some of the non-significant findings of this study relative to significant findings reported in larger cohort studies (e.g., Magson et al. 2020, N *=* 248) that also investigated outcomes of anxiety, depressive symptoms and SWB pre-pandemic and during COVID-19. Missing data may have been due to several reasons. Parental consent was obtained in the weeks prior to data collection and some students may have been absent during the days of the survey. Participation was voluntary and it is possible that although students attended, they may have opted out on the day due to lack of motivation to start or finish the survey. Future multi-site data collection will generate a larger sample pool and mechanisms, such as incentives for participating schools returning a minimum number of surveys, may mitigate the risk of incidental participant drop-out. Future studies incorporating other methods of data collection (e.g., behavioural observation, teacher/peer report) could provide a more complete understanding of the variables under investigation. This could reduce the risk/impact of response biases associated with self-reported measures, which was a limitation in the current study. Demographically, participants from one private co-educational faith-based school are not representative of the diversity of Australian adolescents. Moreover, as low family income is an identified risk factor for mental health issues during this pandemic era [12], it is questionable whether there is a proportional representation of students from this demographic background in the participating school. Thus, it is recommended future studies recruit students from across different school types (public, private, faith-based and non-faith based) and geographical locations (i.e., rural and urban) to ensure the diversity of the general population is represented. It is further proposed studies include different year level cohorts to test potential differences in the presentation of PsyCap (HERO combined and individual constructs), mental health and subjective wellbeing across age, gender, and time. This is particularly important given the relatively short follow-up interval of the current study early in the pandemic when student mental health symptoms (as indexed by the RCADS-SV) appear relatively stable. Therefore, longitudinal studies of longer-term and multiple follow-up timepoints are needed to determine the lasting impacts of COVID-19 on student mental health and wellbeing. Due to the shared variance between HERO constructs, future research utilising structural equation modelling may serve to increase the construct validity of PsyCap and provide a better understanding of how the individual HERO components contribute to PsyCap and student mental health and wellbeing outcomes. Furthermore, school-based interventions aimed at developing the PsyCap resources of hope, efficacy, resilience and optimism (HERO) in students may be useful in reducing mental health symptoms and promoting SWB during challenging times such as a worldwide pandemic.

## Summary

The present study contributes to the emergent empirical literature on youth mental health and SWB in the COVID-19 era. Specifically, it attends to the research gap by providing longitudinal assessments of pre-pandemic and pandemic levels of youth mental health symptoms and SWB and the influence of baseline PsyCap on these outcomes during the early months of an unprecedented global stressor. Overall, the present findings suggest that the roles of positive psychological resources, mental health symptoms and SWB warrant further attention to understand young people’s adjustment to the pandemic and life beyond COVID-19.

## Data Availability

Due to the nature of this research, participants of this study did not agree for their data to be shared publicly, so supporting data is not available.
